# Transactions of the College of Physicians of Philadelphia

**Published:** 1855-03

**Authors:** 


Transactions of the College of Physicians of Philadelphia.—Dr. Wilson presented a specimen of the plant and of the essential oil of the *Erigeron Philadelphicum*, and read the following notes of several cases of uterine hemorrhage successfully treated by the the use of the latter:

*Mr. President:* I have here a specimen of *Erigeron Philadelphicum.* It is an indigenous plant, growing abundantly in the fields around about Philadelphia, and has been used as a remedial agent for a long time, and, of course, is familiar to the members of the College. It is, however, the oil of the *Erigeron Philadelphicum* to which I wish to call the attention of the College for a few minutes. It is very extensively used and highly extolled by the eclectic physicians, as a remedy in various diseases—diarrhœa, dysentery, hæmoptysis, hæmatemesis, menorrhagia, uterine hemorrhage, &c., &c.

It is of a light straw color; is very limpid; has a peculiar aromatic, not unpleasant odor, which is extremely persistent; its taste is peculiar, mild, and not very pungent. Professor Proctor, who has examined it, informs us that its density is very low, being 0·845; it is very inflammable. It begins to boil at 310° Fahr., and continues to increase in temperature to 365°; showing that there are two volatile oils in association. The amount of oil yielded is extremely small.

My attention was called to it in January last, by Dr. A. C. Bournonville, as a remedy in uterine hemorrhage. He informed me that he had used it in some few cases, and wished me to try it. I have prescribed it in many cases, and very generally with decided advantage. The following are a few of those of which I kept notes:—

1. Mrs. E. W., residing in Adams street, east from Thirteenth street. I visited her in January, 1854. She was 44½ years old, pale, weak, with loss of appetite; scarcely able to be about the house; had been unwell for five weeks, for the last five days very bad. Gave her 5 gtt. every two hours. She was entirely well in thirty hours.

2. Mrs. H. W. had miscarried at the sixth week of utero-gestation; flooding had continued for ten days after the expulsion of the ovum. Gave her 5 gtt. every two hours. She was entirely well in forty eight hours.

3. Miss F. D., St. John street, above Poplar street, aged 21 years; stout, muscular woman; pale, weak, loss of appetite, &c.; had been unwell for thirteen weeks, using from four to six napkins daily during most of the time. Gave her 5 gtt every two hours during the day. She was entirely well in twelve days. She went about her house during the treatment, but was restricted to light work.

4. Mrs. S., South Fourth street, had miscarried at the tenth week; was a large, stout, healthy woman, about 37 years old; the hemorrhage was very much controlled by the use of this medicine.

5. Mrs. R., Marshall street, above Master, had been unwell for ten weeks, many days so bad that she was compelled to go to bed. Gave 5 gtt. every two hours. She was entirely well in four days.

I have also prescribed it in some cases of irritation of the bladder, with great relief.

---

Dr. Coates made some remarks on the utility of milfoil (*achillæa millefolium*), in hemorrhage.

Dr. Coates stated that he had used an infusion of achillæa millefolium, sometimes popularly called milfoil and yarrow, with material advantage, in several cases of hemorrhage; two from the kidneys, three (in the same subject) from the uterus, and one from the lungs. He apprehended that the vegetable astringents were too little employed in this country. The infusion was taken without encountering any particular repulsiveness; and, as it seemed to him, rather strengthened than disordered digestion. Without a vegetable astringent, he should have most probably employed some more debilitating or deranging agent; for example —the acetate of lead. The formula was ℥ss. of the herb, to ℔i. of hot water; to digest twenty minutes, covered; the result to be taken cold, in the dose of half a teacupful every two hours.

---

Dr. Yardly presented a thin caoutchouc bottle, furnished with a stop-cock and detached tube, which he had used successfully to arrest uterine hemorrhage by inflating it within the vagina.

Dr. Meigs referred to the use of a similar instrument by Dr. Karl Braun, of Vienna, for the purpose of overcoming rigidity of the os uteri by dilating the vagina.

